# New jatropholane-type diterpenes from *Jatropha curcas cv. Multiflorum* CY Yang

**DOI:** 10.1007/s13659-013-0031-x

**Published:** 2013-05-29

**Authors:** Yuan-Feng Yang, Jie-Qing Liu, Lei Shi, Zhong-Rong Li, Ming-Hua Qiu

**Affiliations:** 1 State Key Laboratory of Phytochemistry and Plant Resources in West China, Kunming Institute of Botany, Chinese Academy of Sciences, Kunming, 650201 China; 2 University of Chinese Academy of Sciences, Beijing, 100049 China; 3 Yunnan Agricultural University, Kunming, 650201 China

**Keywords:** *Jatropha curcas cv. Multiflorum*, Euphorbiaceae, diterpenes, antibacterial

## Abstract

Three new jatropholane-type diterpenoids, jatropholones C-E (**1–3**), along with seven other known compounds, including sikkimenoid B (**4**), jatrophaldehyde (**5**), *epi*-jatrophaldehyde (**6**), *epi*-jatrophol (**7**), jatrophol (**8**), jatropholone A (**9**), and jatropholone B (**10**), were isolated from the roots of a natural cultivar of *Jatropha curcas* (*J. curcas cv. Multiflorum* CY Yang). The structural elucidations of **1–3** were accomplished by extensive NMR analysis. Compounds **4, 6**, and **8** demonstrated inhibition activity against the microorganisms with the MIC values from 0.10 to 0.18 mg/mL.

**Electronic Supplementary Material:**

Supplementary material is available for this article at 10.1007/s13659-013-0031-x and is accessible for authorized users.

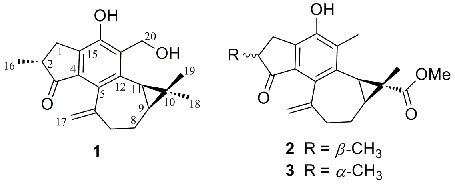

## Introduction

The genus *Jatropha* belonging to the family Euphorbiaceae contains a number of traditionally important medicinal plants. Approximately 170 species are found in tropical Asia and Africa, while only three common cultivated and three wild species (*J. curcas*, *J. podagrica*, and *J. multifida*), together with two cultivated species (*J. gossypifolia* and *J. integerrima*) distributed in China. *J. curcas* and *J. podagrica*, have been used as Chinese traditional medicines for the treatment of injuries swelling, fracture, skin itching, eczema, and acute gastroenteritis embolism[1]. Recently, two natural cultivars of *J. curcas* (*J. curcas cv. Multiflorum* CY Yang and *J. nigroviensrugosus* CY Yang) were discovered in the Yuanjiang River, Yunnan province and had been registered as new cultivars[2].

The chemical constituents from this genus consisted of diterpenoids, sesquiterpenoids, lignans, coumarins, flavonoids, and some others, of which diterpenoids were the main chemical components with diverse skeletons, including jatropholane, lathyrane, and rhamnofolane type[3]. Up until recently, more than 65 diterpenoids were identified from this genus[4]. Some of these have been proven to have the effects of anti-inflammatory[5], antiproliferation[6,7], antiplasmodial[8], anti-tuberculosis[9], and antitumor[9–11].

*J. curcas cv. Multiflorum*, as one of two natural cultivars of

*J. curcas*, had the morphological characteristics as follows: I) It can blossom many times in a year with a number of flowers, fruits, and branches; II) It can blossom at the beginning of germination after cutting branches; III) Compared with *J. curcas*, the most striking trait is that the production of seed of the plant is higher than that of *J. curcas*[12].

Our previous phytochemical investigation on *J. curcas* from different geographic regions has resulted in the identification of jatropholane and rhamnofolane diterpenes[13], and of sesquiterpenes[14]. However to date, no phytochemical research has been carried out on *J. curcas cv. Multiflorum*. Therefore, in order to identify the chemical components and examine for structurally diverse and biologically significant compounds, an initial investigation on the chemical compositions of *J. curcas cv. Multiflorum* was undertaken.

*To whom correspondence should be addressed. E-mail: mhchiu@mail.kib.ac.cn

**Table 1.** $^1H$ **and** $^{13}C$ **NMR spectroscopic data of 1–3 (600 and 150 MHz;** $CDCl_3$**;** $\delta$ **in ppm)**

| | **1** | | **2** | | **3** | |
|---|---|---|---|---|---|---|
| pos. | $\delta_H$ (*J* in Hz) | $\delta_C$ | $\delta_H$ (*J* in Hz) | $\delta_C$ | $\delta_H$ (*J* in Hz) | $\delta_C$ |
| 1$\alpha$ | 3.25 (dd, 17.2, 7.8) | 30.3 t | 3.27 (dd, 16.5, 8.1) | 30.2 t | 3.20 (m) | 30.0 t |
| 1$\beta$ | 2.58 (overlap) | | 2.51 (dd, 16.5, 4.5) | | 2.54 (m) | |
| 2 | 2.60 (overlap) | 42.4 d | 2.73 (overlap) | 42.5 d | 2.73(overlap) | 42.3 d |
| 3 | | 208.5 s | | 207.6 s | | 207.8 s |
| 4 | | 139.7 s | | 137.2 s | | 137.7 s |
| 5 | | 133.9 s | | 134.4 s | | 134.4 s |
| 6 | | 145.4 s | | 144.7 s | | 144.2 s |
| 7 | 2.63 (overlap) | 33.3 t | 2.72 (overlap) | 33.5 t | 2.72 (overlap) | 33.5 t |
| 8$\alpha$ | 1.81 (m) | 21.3 t | 1.86 (overlap) | 20.7 t | 1.86 (overlap) | 20.7 t |
| 8$\beta$ | 0.84 (m) | | 1.00 (m) | | 0.96 (m) | |
| 9 | 0.94 (m) | 26.2 d | 1.90 (overlap) | 28.0 d | 1.89 (overlap) | 28.0 d |
| 10 | | 19.6 s | | 26.7 s | | 26.7 s |
| 11 | 1.51 (d, 8.7) | 27.3 d | 2.58 (d, 9.5) | 30.4 d | 2.59 (d, 9.5) | 30.4 d |
| 12 | | 134.7 s | | 134.2 s | | 134.3 s |
| 13 | | 130.0 s | | 130.1 s | | 130.3 s |
| 14 | | 153.2 s | | 150.1 s | | 150.1 s |
| 15 | | 133.3 s | | 132.9 s | | 132.1 s |
| 16 | 1.29 (d, 7.4) | 17.0 q | 1.29 (d, 7.3) | 15.8 q | 1.30 (d, 7.5) | 17.0 q |
| 17a | 5.25 (s) | 115.3 t | 5.25 (s) | 116.0 t | 5.27 (s) | 116.3 t |
| 17b | 4.68 (s) | | 4.71 (s) | | 4.75 (s) | |
| 18 | 1.20 (s) | 28.0 q | | 176.2 s | | 176.2 s |
| 19 | 0.81 (s) | 16.5 q | 1.05 (s) | 10.3 q | 1.04 (s) | 10.2 q |
| 20 | 5.01 (d, 13.5) | 62.1 d | 2.21 (s) | 13.0 q | 2.22 (s) | 13.0 q |
| | 5.05 (d, 13.5) | | | | | |
| 19-COO*CH*$_3$ | | | 3.74 (s) | 52.3 q | 3.74 (s) | 52.3 q |

Our first-time phytochemical study on the plant collected in Xishuangbanna Tropical Botanical Garden, Chinese Academy of Sciences, led to the isolation of three new jatropholane-type diterpenoids (**1**–**3**), along with seven known jatropholane analogs (**4**–**10**). Their structures were determined by analysis of the spectroscopic data. Compounds **1**–**10** had been evaluated for their cytotoxic activity and compounds **4**, **6**, **8**, and **10** were further tested for their antibacterial effect against *Staphylococcus aureus*, *Escherichia coli*, *Bacillus subtilis*, *Pseudomonas aeruginosa*, and *Staphylococcus epidermidis*. Herein, the isolation, structural elucidation and biological evaluation of these compounds were reported.

## Results and Discussion

The $CHCl_3$-soluble fraction of the methanol extract of the air-dried powdered *J. curcas cv. multiflorum* was repeatedly chromatographed on silica gel, RP-18, and HPLC to yield ten jatropholane-type diterpenes (**1**–**10**). Of them, seven known compounds were identified as sikkimenoid B (**4**)[15], jatrophaldehyde (**5**)[16], *epi*-jatrophaldehyde (**6**)[16], *epi*-jatrophol (**7**)[16], jatrophol (**8**)[17], jatropholone A (**9**)[18], and jatropholone B (**10**)[18] in comparison to their spectroscopic data with those described in the literature.

Compound **1** was assigned the molecular formula $C_{20}H_{24}O_3$, with nine degrees of unsaturation, on the basis of positive HREIMS at *m/z* 312.1949 $[M]^+$ (calcd for $C_{20}H_{24}O_3$, 312.1937). The IR spectrum displayed absorption bands for hydroxyl group (3432 $cm^{-1}$), carbonyl group (1712 $cm^{-1}$), and an exocyclic double bond (1640 and 906 $cm^{-1}$). The $^1H$ and $^{13}C$ NMR spectral data (Table 1) suggests that compound **1** possessed jatropholane-type skeleton comprising a 5/6/7/3 fused ring system, similar to those of jatropholone B (**10**)[18], except for a hydroxymethyl group in **1** instead of the methyl group in **10**. The presence of HMBC correlations from the hydroxymethyl protons at $\delta_H$ 5.01 (d, *J* = 13.5 Hz) and 5.05 (d, *J* = 13.5 Hz) to the carbon signals at $\delta_C$ 134.7 (C-12), 130.0 (C-13), and 153.2 (C-14) indicated that the $CH_2OH$ group was assigned at C-13. The plane structure of **1** could be further confirmed by their COSY and HMBC correlations (Figure 1).

**Figure 1.** $^1H$-$^1H$ COSY ( ▬ ) and key HMBC (→) correlations of **1**

Compound **1** possessed the same skeleton with the compounds reported in the literature[16–18], the orientations of both H-9 and H-11 in them were $\alpha$-orientation and confirmed by X-ray crystallography recently[16], thus, the relative configurations of H-9 and H-11 in **1** were $\alpha$. Moreover, the $\alpha$-orientation of Me-16 was established by comparison of the chemical shifts, splitting patterns, and coupling constants of Me-16 ($\delta_C$ 17.0; $\delta_H$ 1.29, d, *J* = 7.4 Hz) of **1** with those of the literature values (Me-16: $\delta_C$ 17.0; $\delta_H$ 1.34, d, *J* = 7.0 Hz) reported for jatropholone B[18a], which also has an $\alpha$-oriented Me-16 group. Hence, the structure of **1** was deduced and named as jatropholone C.

Compound **2** was isolated as white powder. Its molecular formula was determined to be $C_{21}H_{24}O_4$ by the molecular ion peak at *m/z* 340.1679 (calcd for $C_{21}H_{24}O_4$, 340.1675) in the HREIMS. The $^1H$ and $^{13}C$ NMR data (Table 1) of compound **2** bore a close resemblance to those of jatrophaldehyde (**5**)[16], and the distinction was that the aldehyde group at C-18 in **5** was replaced by a methoxycarbonyl group in **2**. This deduction was further confirmed by the HMBC (Figure 2) correlations from H-9 ($\delta_H$ 1.90), H-11 ($\delta_H$ 2.58), Me-20 ($\delta_H$ 2.21), and the OMe proton ($\delta_H$ 3.74, s) to the ester carbonyl ($\delta_C$ 176.2). The distinct correlations of Me-18 with H-9 and H-11 were observed in the ROESY spectrum of **1**. However, the above-mentioned correlations were not found in the ROESY spectrum of **2**. Additionally, the carbon resonance of Me-19 in **2** was $\delta_C$ 10.3, which was similar to that of $\beta$-oriented Me-19 group ($\delta_C$ 10.1) in compounds **5a**[19]. Thus, the -COOMe group in **2** was of $\alpha$ configuration.

**Figure 2.** Key HMBC (→) correlations of **2**

Compound **3** was found by HREIMS (*m/z* 340.1678 $[M]^+$, calcd for $C_{21}H_{24}O_4$, 340.1675) to have the molecular formula, $C_{21}H_{24}O_4$, the same as that of **2**. Detailed comparison of the $^1H$ and $^{13}C$ NMR spectroscopic data (Table 1) of compounds **3** and **2** found that the chemical shift values between them were almost the same, with a deviation of ~0.5 ppm, respectively. Nevertheless, the chemical shift of Me-16 ($\delta_C$ 17.0) in **3** was remarkably downfield shifted by 1.2 ppm than that of in **2**, assuming that they might be C-2 epimers. The $\beta$-oriented Me-16 ($\delta_C$ 15.8) in **2** was deduced by the similar carbon signal of Me-16 ($\delta_C$ 15.0) in **5**, and the $\alpha$-oriented Me-16 ($\delta_C$ 17.0) in **3** was determined via the analogous carbon signal of Me-16 ($\delta_C$ 16.4) in **6**[16]. Finally, the structures of compounds **2** and **3** were elucidated and named as jatropholone D and jatropholone E, respectively.

Compounds **1**–**10** were evaluated for their cytotoxicity against five human tumor cell lines, including HL-60, SMMC-7721, A549, MCF-7 and SW480. However, no cytotoxicity against above assayed cell lines were observed ($IC_{50}$ > 40 $\mu$M). Compounds **4**, **6**, and **8** demonstrated inhibition activity against the microorganisms with the MIC values from 0.10 to 0.18 mg/mL (Table 2).

## Experimental Section

**General Experimental Procedures.** Optical rotations were measured on a JASCO P-1020 digital polarimeter. UV spectra were obtained in MeOH with a Shimadzu UV-2401 PC spectrophotometer. IR spectra were recorded on a Brucker Tensor 27 infrared spectrophotometer with KBr pellets. ESIMS spectra were performed on an API QSTAR Pulsar spectrometer, while HREIMS were measured on a Waters Autospec Premier P776 mass spectrometer instrument in positive ion mode. 1D and 2D NMR spectra were carried out on Bruker Avance III-600MHz spectrometer. Chemical shifts ($\delta$) were expressed in *ppm* with respect to the TMS resonance. Semi-preparative HPLC was collected on an Agilent 1100 liquid chromatograph with an YMC-PackPro$C_{18}$RS (YMC, 250 × 10 mm, 5 $\mu$m) column. Column chromatography was performed with silica gel (200–300 mesh; Qingdao Marine Chemical Co., Ltd., Qingdao, China), Lichroprep RP-18 silica gel (40–63 $\mu$m, Merck, Darmstadt, Germany), Sephadex LH-20 (20–150 $\mu$m, Pharmacia); Fractions were monitored by TLC under UV light, and spots were visualized by heating silica gel plates sprayed with 10% aqueous $H_2SO_4$ in EtOH. All solvents including petroleum ether (60–90 °C) were distilled prior to use.

**Plant Material.** The plant materials were collected in Xishuangbanna Tropical Botanical Garden, Chinese Academy of Sciences, in July 2012 and identified by Associate Professor Cheng-Yuan Yang. A voucher specimen (No. KIB 20120701) has been deposited at the State Key Laboratory of Phytochemistry and Plant Resources in West China, Kunming Institute of Botany, Chinese Academy of Sciences.

**Extraction and Isolation.** The air-dried roots (4.5 kg) were coarsely powdered and extracted with MeOH (3 × 18 L, each 4 h) under reflux at 55 °C. The combined MeOH solution was evaporated in vacuo to produce a dark brown residue, which was suspended in water and then extracted with $CHCl_3$. The $CHCl_3$-soluble layer (168.5 g) was subjected to column chromatography (CC) over silica gel with acetone/petroleum ether (1:15, 1:10, 1:5, 1:2, 1:1) as elution to afford 5 fractions (Fr.1–Fr.5). Fr.2 (20.0 g) was chromatographed further on RP-18 CC with MeOH/$H_2O$ (55%, 62%, and 75%) to obtain Fr.2.1 (6 g), Fr.2.2 (4 g), and Fr.2.3 (7 g). Fr.2.2 was decolorized on Sephadex LH-20 with MeOH to obtain a brown gum (5.5 g). The gum was further submitted to semi-preparative HPLC (3 mL/min, UV detection at $\lambda_{max}$ = 254 nm, $CH_3CN/H_2O$, 60:40) to gain compounds **2** (2.2 mg), **3** (1.7 mg), **5** (20.0 mg), and **6** (23.0 mg). Compounds **9** (32.0 mg) and **10** (28.5 mg) were obtained from fraction Fr.2.2 using a silica gel column ($CHCl_3$/petroleum ether, 1:5). Fr.2.3 was further treated in sephadex LH-20 with MeOH and semi-preparative HPLC ($CH_3OH/H_2O$, 70:30) to gain compound **4** (1.5 mg). Fr.3 (18 g, acetone/petroleum ether, 1:5) was fractionated first on RP-18 with a gradient elution with MeOH/$H_2O$ (45%, 60%, and 75%) to yield subfractions Fr.3.1−Fr.3.3. Subsequently, Fr.3.2 (5 g) was further fractionized on a silica gel column eluting with acetone/petroleum ether (1:8) to afford subfractions Fr.3.2.1 (30 mg) and Fr.3.2.2 (240 mg). Fr.3.2.1 was further purified by semi-preparative HPLC (3 mL/min, UV detection at $\lambda_{max}$ = 254 nm, $CH_3CN/H_2O$, 50:50) to yield **1** (1.7 mg). Further separation of Fr.3.2.2 on silica gel eluting with acetone/petroleum ether (1:8, 1:4) gave compounds **7** (20 mg) and **8** (30 mg).

**Jatropholone C (1):** white amorphous powder; $[\alpha]_D^{25}$ − 50.5 (*c* 0.150, $CHCl_3$); UV ($CHCl_3$) $\lambda_{max}$ (log $\varepsilon$): 314 (3.73), 264 (4.17), 240 (4.26) nm; IR (KBr) $\nu_{max}$ 3432, 1712, 1640, 906 $cm^{-1}$; $^1H$ and $^{13}C$ NMR data, see Table 1; ESIMS *m/z* 335 $[M + Na]^+$; HREIMS *m/z* 312.1949 $[M]^+$ (calcd for $C_{20}H_{24}O_3$, 312.1937).

**Jatropholone D (2):** white amorphous powder; $[\alpha]_D^{17}$ + 15.3 (*c* 0.150, $CHCl_3$); UV ($CHCl_3$) $\lambda_{max}$ (log $\varepsilon$): 314 (3.85), 265 (4.28), 240 (4.48) nm; IR (KBr) $\nu_{max}$ 3428, 2955, 2924, 1717, 1696, 1599, 1464 $cm^{-1}$; $^1H$ and $^{13}C$ NMR data, see Table 1; ESIMS *m/z* 363 $[M + Na]^+$; HREIMS *m/z* 340.1679 $[M]^+$ (calcd for $C_{21}H_{24}O_4$ $[M]^+$, 340.1675).

**Table 2. Antibacterial data of compounds 4, 6, 8, and 10**

| compound | zones of inhibition (mm)/MIC (mg/mL) | | | | |
|---|---|---|---|---|---|
| | *E. coli* | *S. aureus* | *P. aeruginosa* | *S. epidermidis* | *B. subtilis* |
| **4** | 22.15/0.11 | 27.98/0.09 | 21.96/0.13 | 17.96/0.15 | 22.89/0.14 |
| **6** | 24.67/0.11 | 27.85/0.10 | 24.32/0.10 | 25.91/0.08 | 23.56/0.12 |
| **8** | 20.44/0.18 | 19.48/0.18 | 20.30/0.19 | 17.18/0.17 | 20.52/0.18 |
| **10** | 19.23/>0.2 | 17.35/>0.2 | 15.44/>0.2 | 14.84/>0.2 | 14.34/>0.2 |
| DMSO | 13.16/ | 14.24/ | 13.32/ | 11.38/ | 13.24/ |

**Jatropholone E (3):** white amorphous powder; $[\alpha]^{17}_{D}$ + 33.7 ($c$ 0.100, $CHCl_3$); UV ($CHCl_3$) $\lambda_{max}$ (log $\varepsilon$): 313 (3.63), 268 (4.07), 241 (4.26) nm; IR (KBr) $\nu_{max}$ 3422, 2965, 2925, 1638, 1464 $cm^{-1}$; $^1H$ and $^{13}C$ NMR data, see Table 1; ESIMS $m/z$ 363 $[M + Na]^+$; HREIMS $m/z$ 340.1678 $[M]^+$ (calcd for $C_{21}H_{24}O_4$ $[M]^+$, 340.1675).

**Cytotoxicity Assay.** Compounds **1–10** were evaluated for their cytotoxicity against several human tumor cell lines, including breast cancer MCF-7, hepatocellular carcinoma SMMC-7721, human myeloid leukemia HL-60, lung cancer A-549, and colon cancer SW480 by the MTT method[13] as previously reported.

**Antibacterial Assays.** The microorganisms employed in the assay were: *S. aureus* (ATCC 25923), *E. coli* (ATCC 25922), *B. subtilis* (ATCC 11060), *P. aeruginosa* (CMCC 10104), and *S. epidermidis* (CMCC 26069). To the well-prepared agar plates, microbial cells along with the compounds were suspended in Mueller Hinton broth at the density of $10^8$ CFU/mL and incubated at 37 °C for 18 h under aerobic conditions. The compounds were dissolved in DMSO to give six different concentrations of 1 $\mu$g/mL, 3 $\mu$g/mL, 9 $\mu$g/mL, 27 $\mu$g/mL, 81 $\mu$g/mL and 243 $\mu$g/mL, added 200 $\mu$L to oxford cup with the diameter of 8 mm. The blank controls of microbial culture were incubated with limited DMSO under the same conditions. The DMSO was determined not to be toxic at a limited amount under the experimental conditions. The controls comprised the inoculated growth medium without test agents, and sample blanks in growth medium only. The plates were incubated for 24 h at 37 °C. The data was obtained through determining the inhibition zone diameters with oxford cup. The MIC value of each compound was determined using a microdilution assay in sterile 96-well microtiter plates[20].

## Electronic Supplementary Material

Supplementary material is available in the online version of this article at http://dx.doi.org/10.1007/s13659-013-0031-x and is accessible for authorized users.

## Acknowledgments

The authors are sincerely grateful to Prof. Cheng-Yuan Yang of the Xishuangbanna Tropical Botanical Garden, Chinese Academy of Sciences, for the identification of the plant. This work was financially supported by the Key Projects of the Chinese Ministry of Science and Technology (2007BAD32B01-03 and SB2007FY400), the National Natural Science Foundation of China (81202437) and the National Knowledge Innovation Program of Chinese Academy of Sciences (KSCX2-YW-G-038).

**Open Access** This article is distributed under the terms of the Creative Commons Attribution License which permits any use, distribution, and reproduction in any medium, provided the original author(s) and source are credited.

## Electronic supplementary material

Supplementary material, approximately 1.07 MB.
