# Supplementary material for: New jatropholane-type diterpenes from Jatropha curcas cv. Multiflorum CY Yang
Source: Nat Prod Bioprospect. 2013 May 29;3(3):99–102. doi: 10.1007/s13659-013-0031-x (PMC4131669; doi:10.1007/s13659-013-0031-x)
Supplement: Supplementary file 1 — Supplementary material, approximately 1.07 MB. [file 13659_2013_31_MOESM1_ESM.pdf]

Electronic Supplementary Material

New jatropholane-type diterpenes from *Jatropha curcas cv. Multiflorum* CY Yang

Yuan-Feng YANG,[a,b] Jie-Qing LIU,[a] Lei SHI,[a,c] Zhong-Rong LI,[a] and Ming-Hua QIU[a,b,]*

[a]State Key Laboratory of Phytochemistry and Plant Resources in West China, Kunming Institute of Botany, Chinese Academy of Sciences, Kunming 650201, China

[b]University of Chinese Academy of Sciences, Beijing 100049, China

[c]Yunnan Agricultural University, Kunming 650201, China

Received 2 April 2013; Accepted 3 May 2013

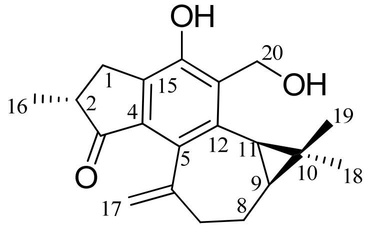

1

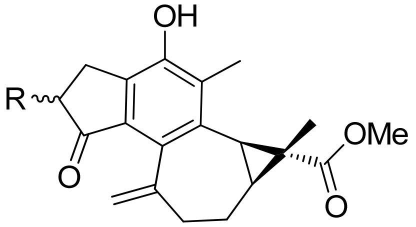

2 R = β-CH_3
3 R = α-CH_3

Structures of compounds **1**–**3**

*To whom correspondence should be addressed. E-mail: mhchiu@mail.kib.ac.cn

Legends for Electronic Supplementary Material Figures

Fig. 1S ^{1}H NMR spectrum of compound **1** ($CDCl_3$, 600 MHz).

Fig. 2S ^{13}C NMR spectrum of compound **1** ($CDCl_3$, 150 MHz).

Fig. 3S HMBC spectrum of compound **1**.

Fig. 4S ^{1}H-^{1}H COSY spectrum of compound **1**.

Fig. 5S HSQC spectrum of compound **1**.

Fig. 6S ROESY spectrum of compound **1**.

Fig. 7S ^{1}H NMR spectrum of compound **2** ($CDCl_3$, 600 MHz).

Fig. 8S ^{13}C NMR spectrum of compound **2** ($CDCl_3$, 150 MHz).

Fig. 9S HMBC spectrum of compound **2**.

Fig. 10S ^{1}H-^{1}H COSY spectrum of compound **2**.

Fig. 11S HSQC spectrum of compound **2**.

Fig. 12S ROESY spectrum of compound **2**.

Fig. 13S ^{1}H NMR spectrum of compound **3** ($CDCl_3$, 600 MHz).

Fig. 14S ^{13}C NMR spectrum of compound **3** ($CDCl_3$, 150 MHz).

Fig. 15S HMBC spectrum of compound **3**.

Fig. 16S ^{1}H-^{1}H COSY spectrum of compound **3**.

Fig. 17S HSQC spectrum of compound **3**.

Fig. 18S ROESY spectrum of compound **3**.

Compound **1**

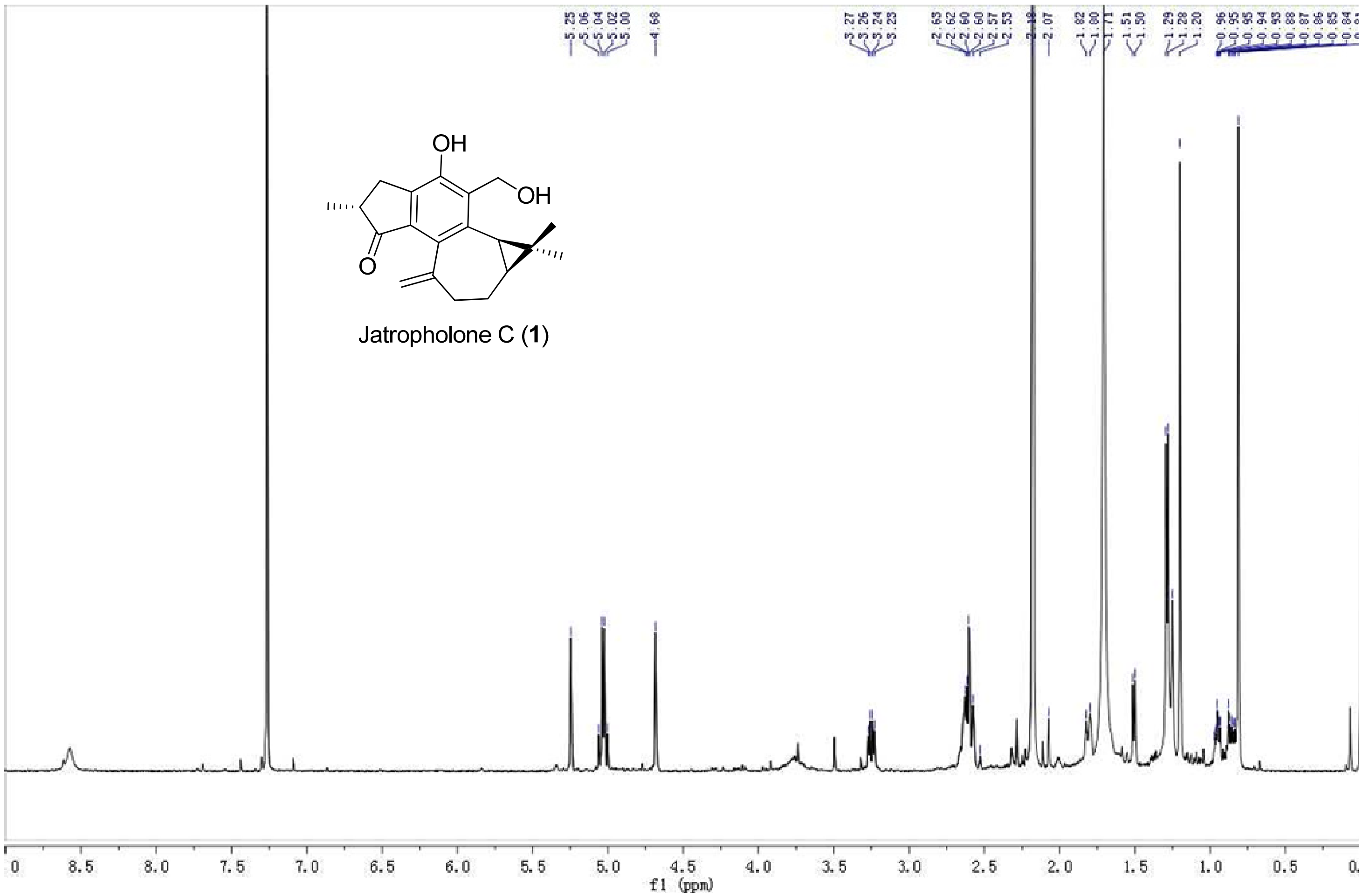

Fig. 1S ^{1}H NMR spectrum of compound **1** ($CDCl_3$, 600 MHz).

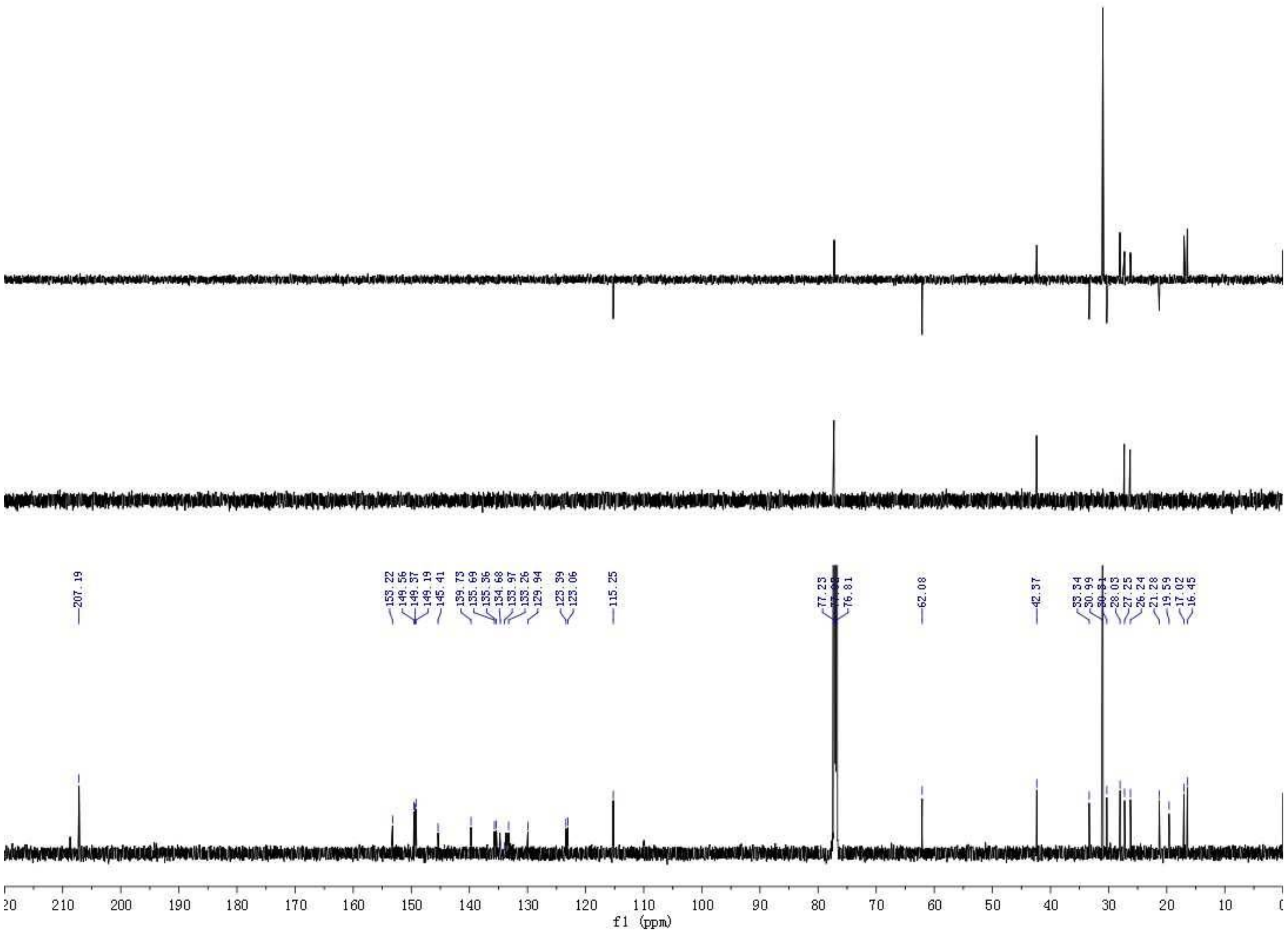

Fig. 2S ^{13}C NMR spectrum of compound **1** ($CDCl_3$, 150 MHz).

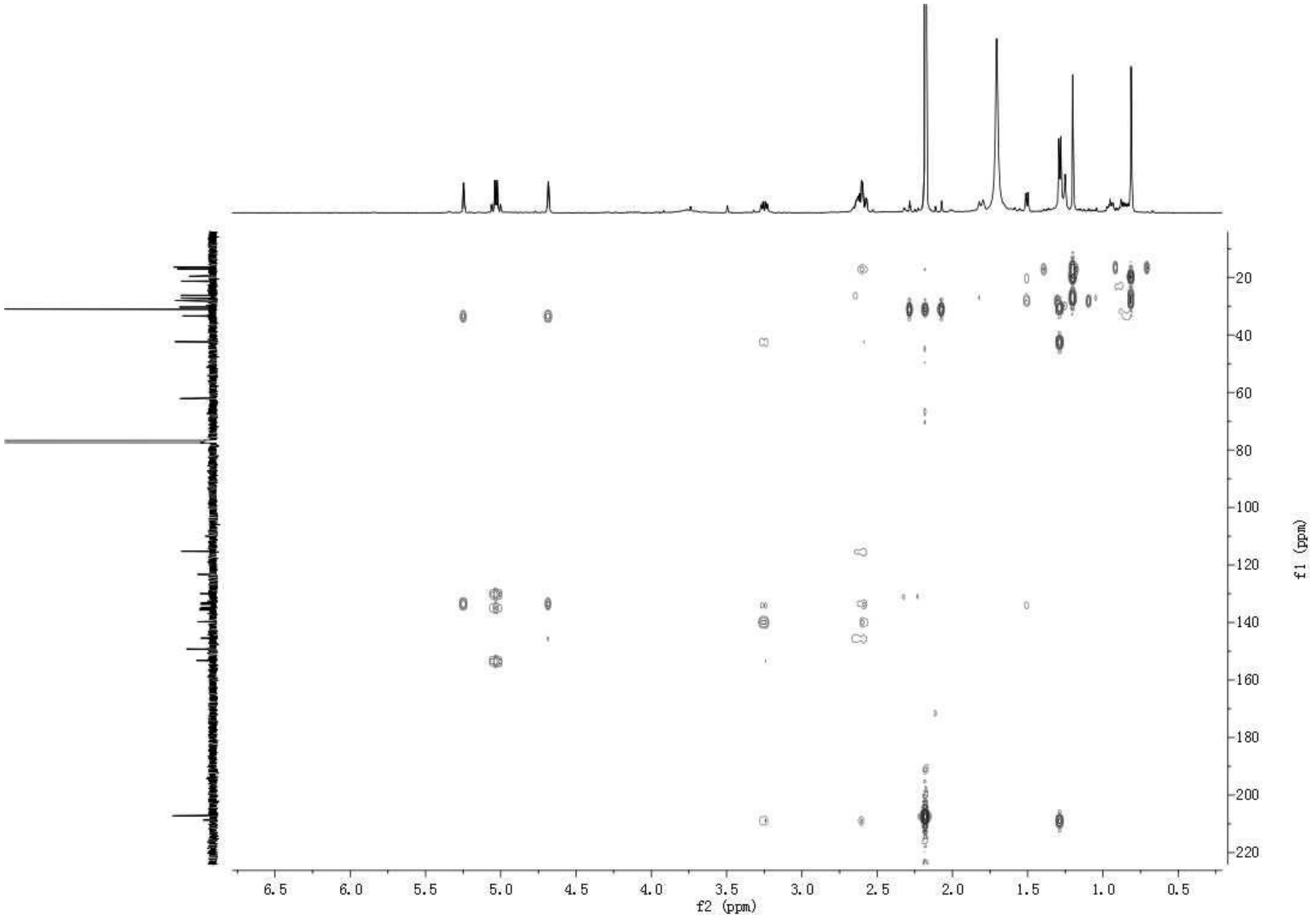

Fig. 3S HMBC spectrum of compound **1**.

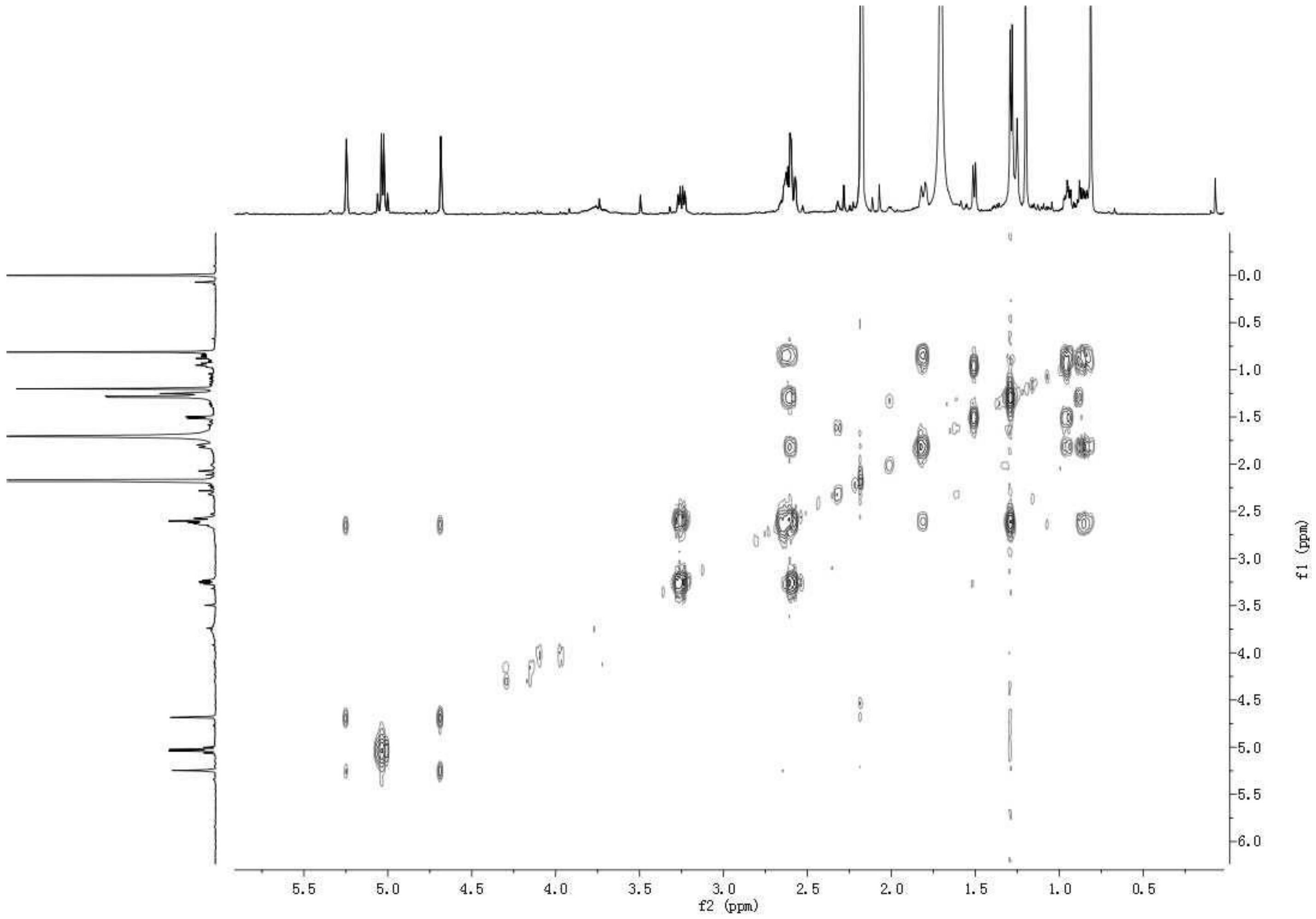

Fig. 4S ^{1}H-^{1}H COSY spectrum of compound **1**.

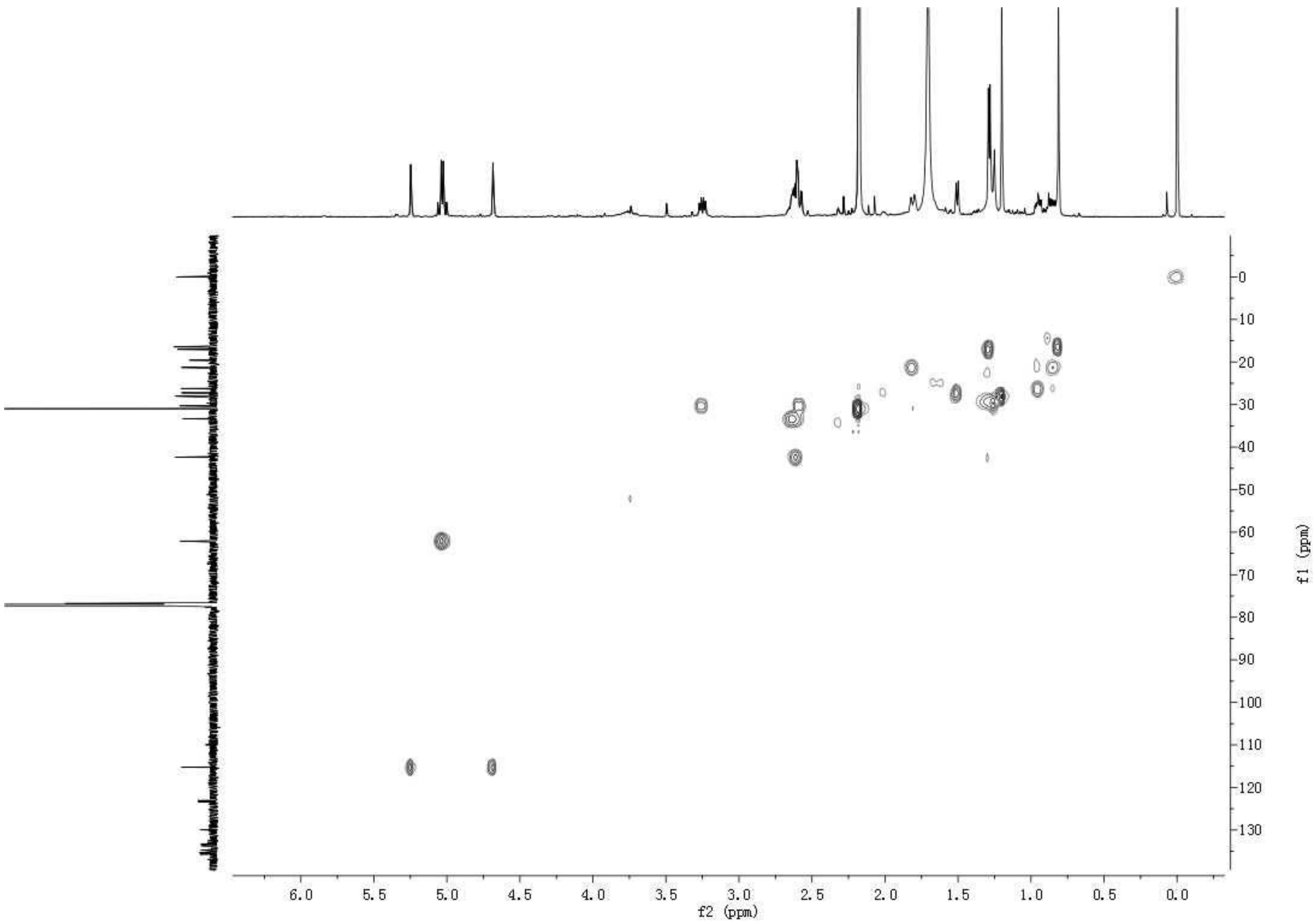

Fig. 5S HSQC spectrum of compound **1**.

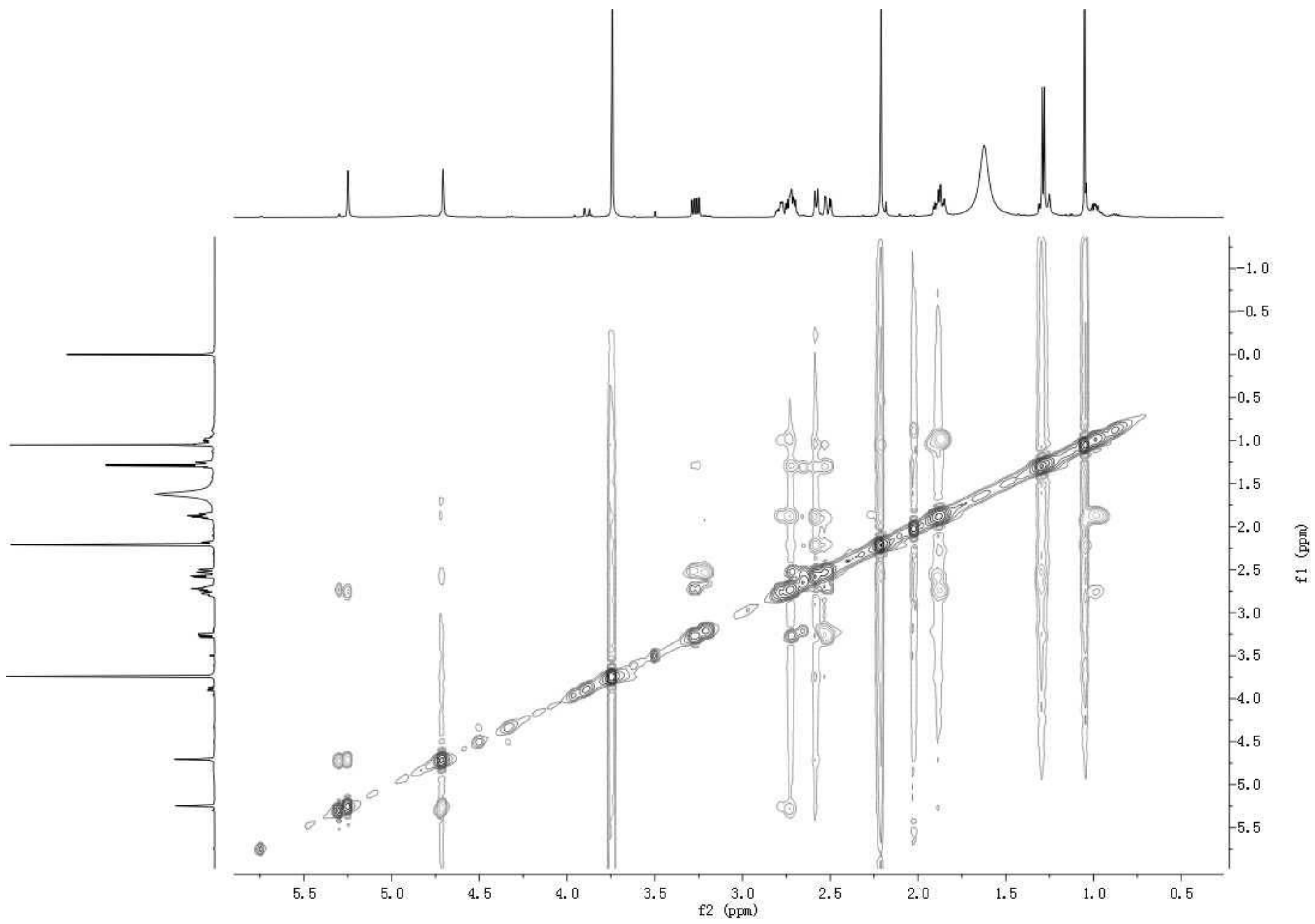

Fig. 6S ROESY spectrum of compound **1**.

Compound **2**

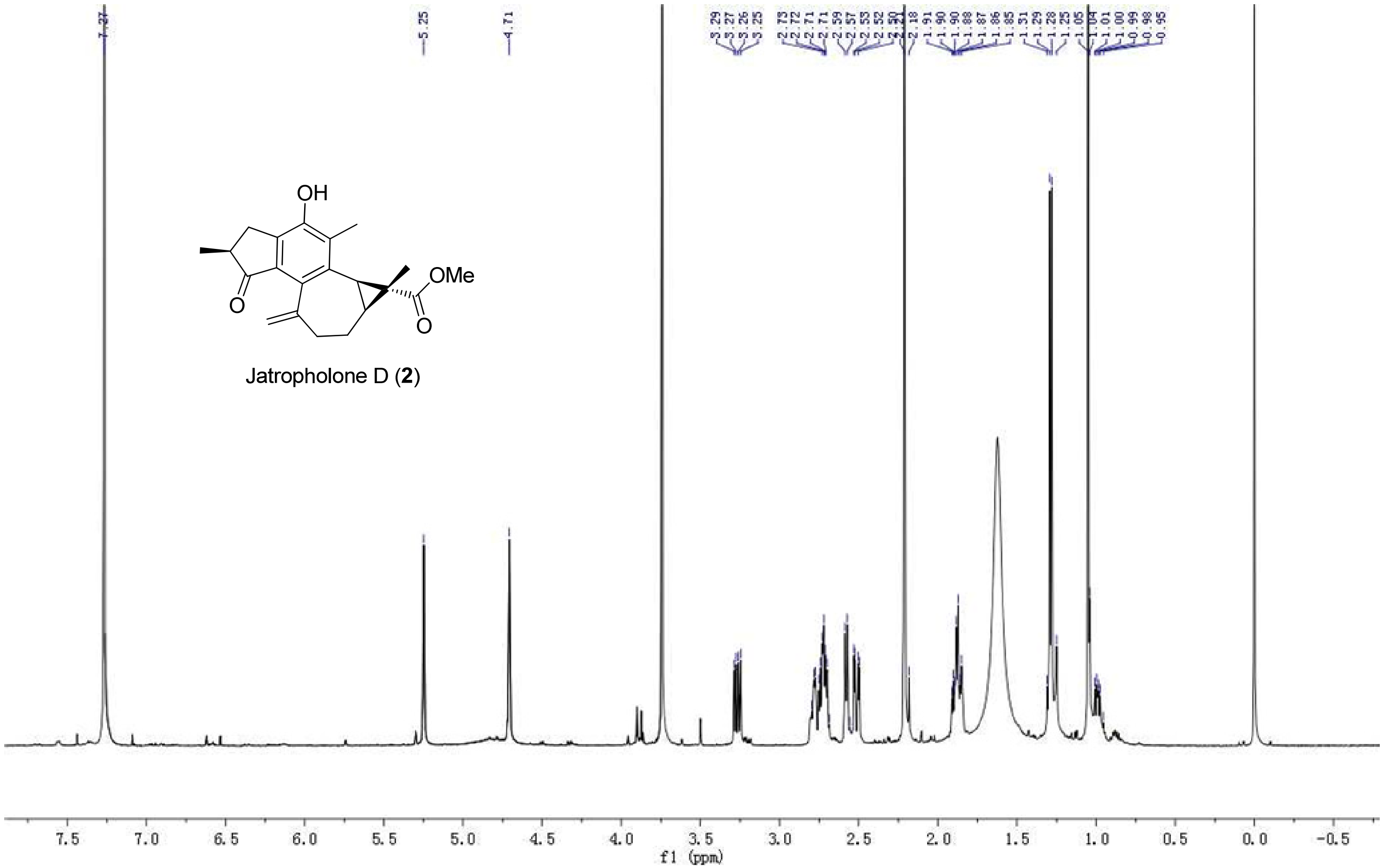

Fig. 7S ^{1}H NMR spectrum of compound **2** ($CDCl_3$, 600 MHz).

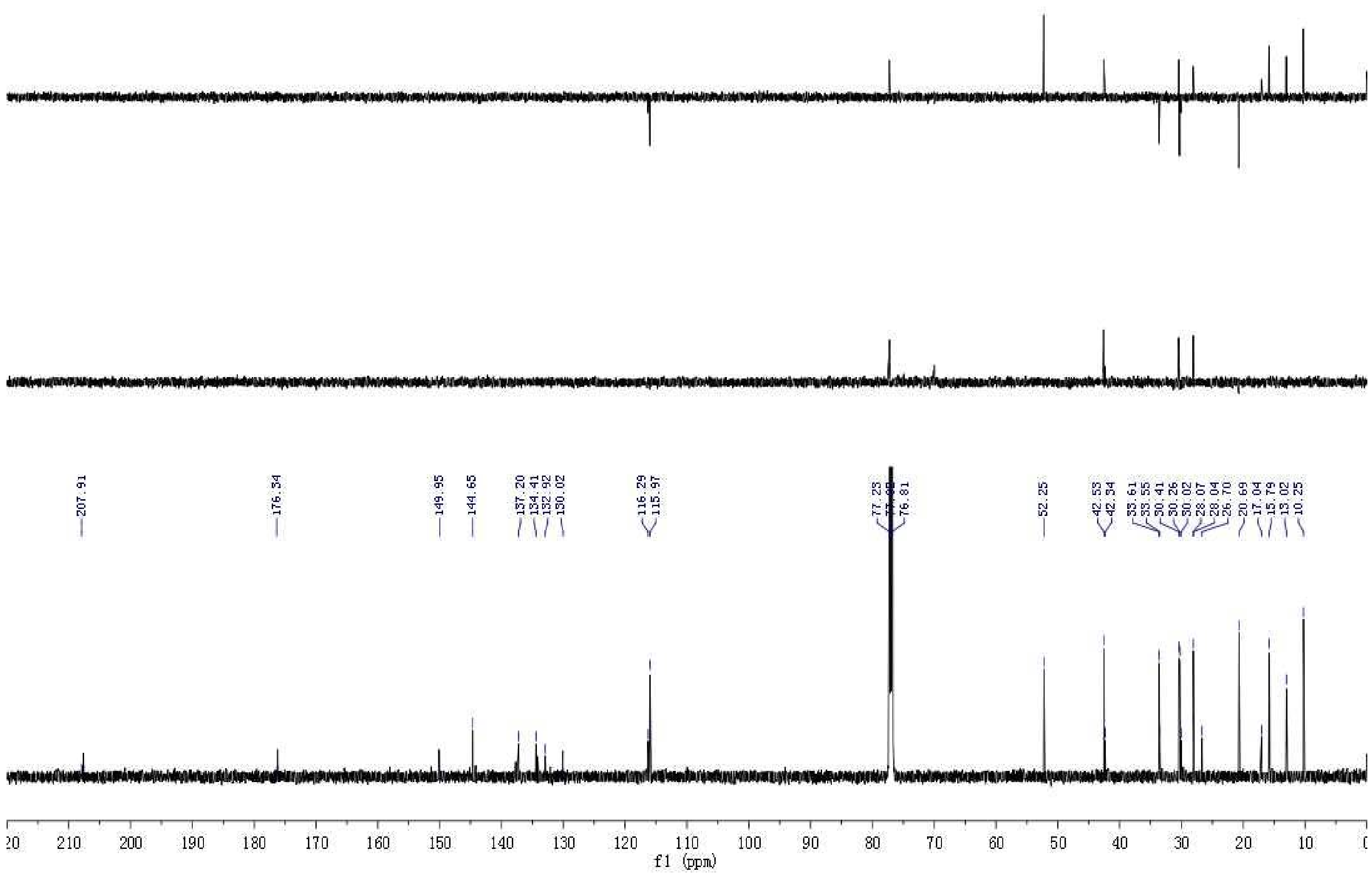

Fig. 8S ^{13}C NMR spectrum of compound **2** ($CDCl_3$, 150 MHz).

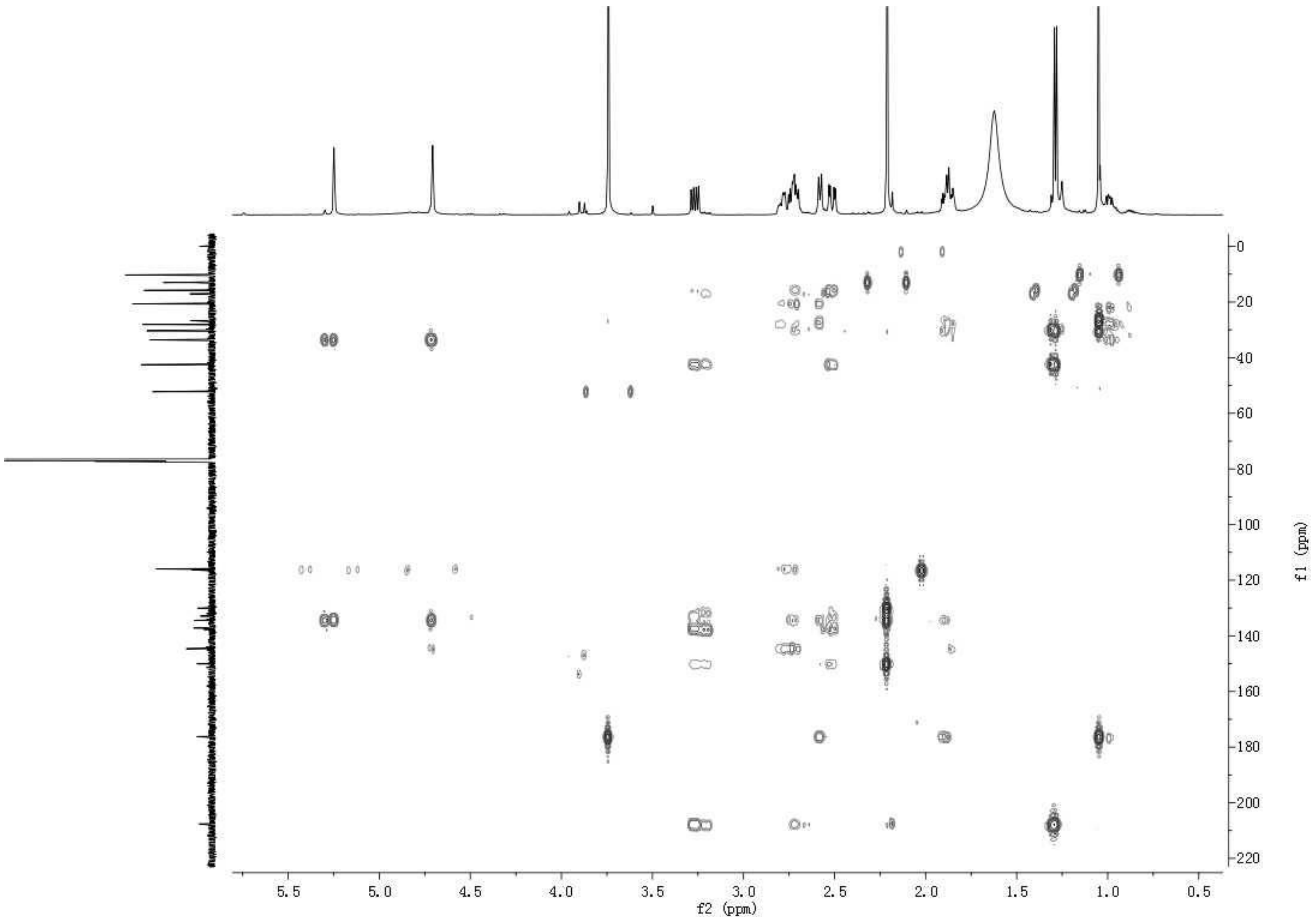

Fig. 9S HMBC spectrum of compound **2**.

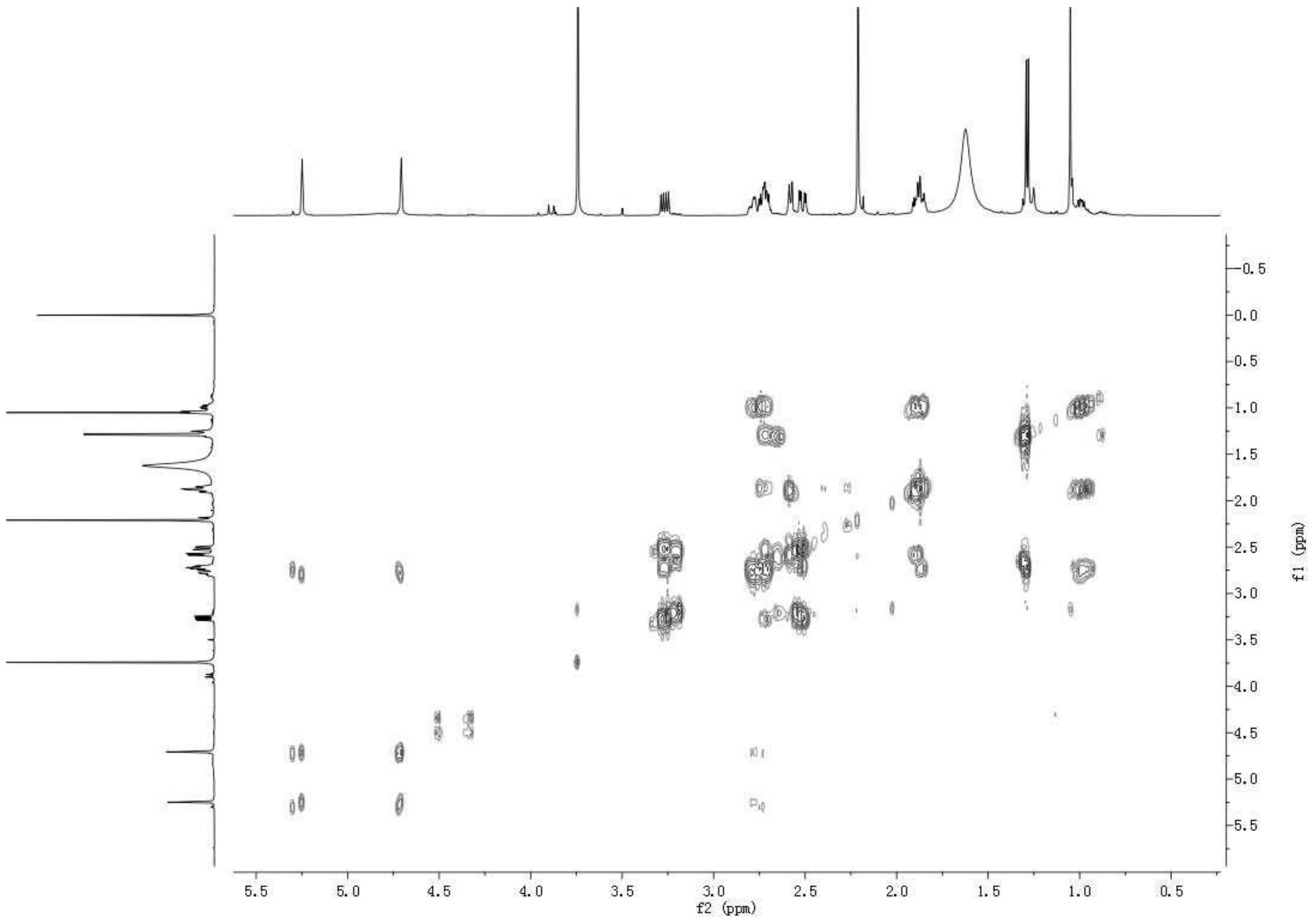

Fig. 10S ^{1}H-^{1}H COSY spectrum of compound **2**.

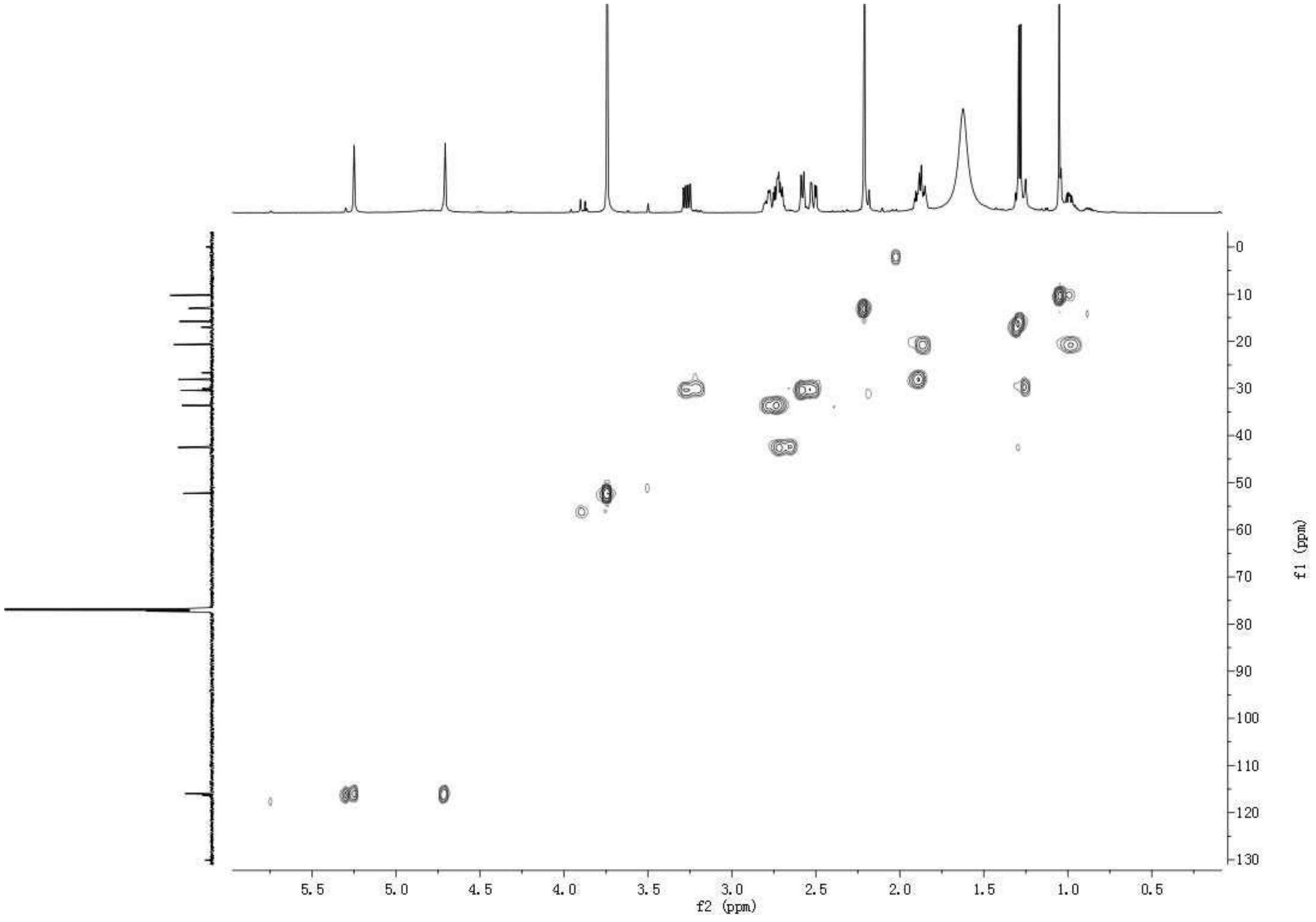

Fig. 11S HSQC spectrum of compound **2**.

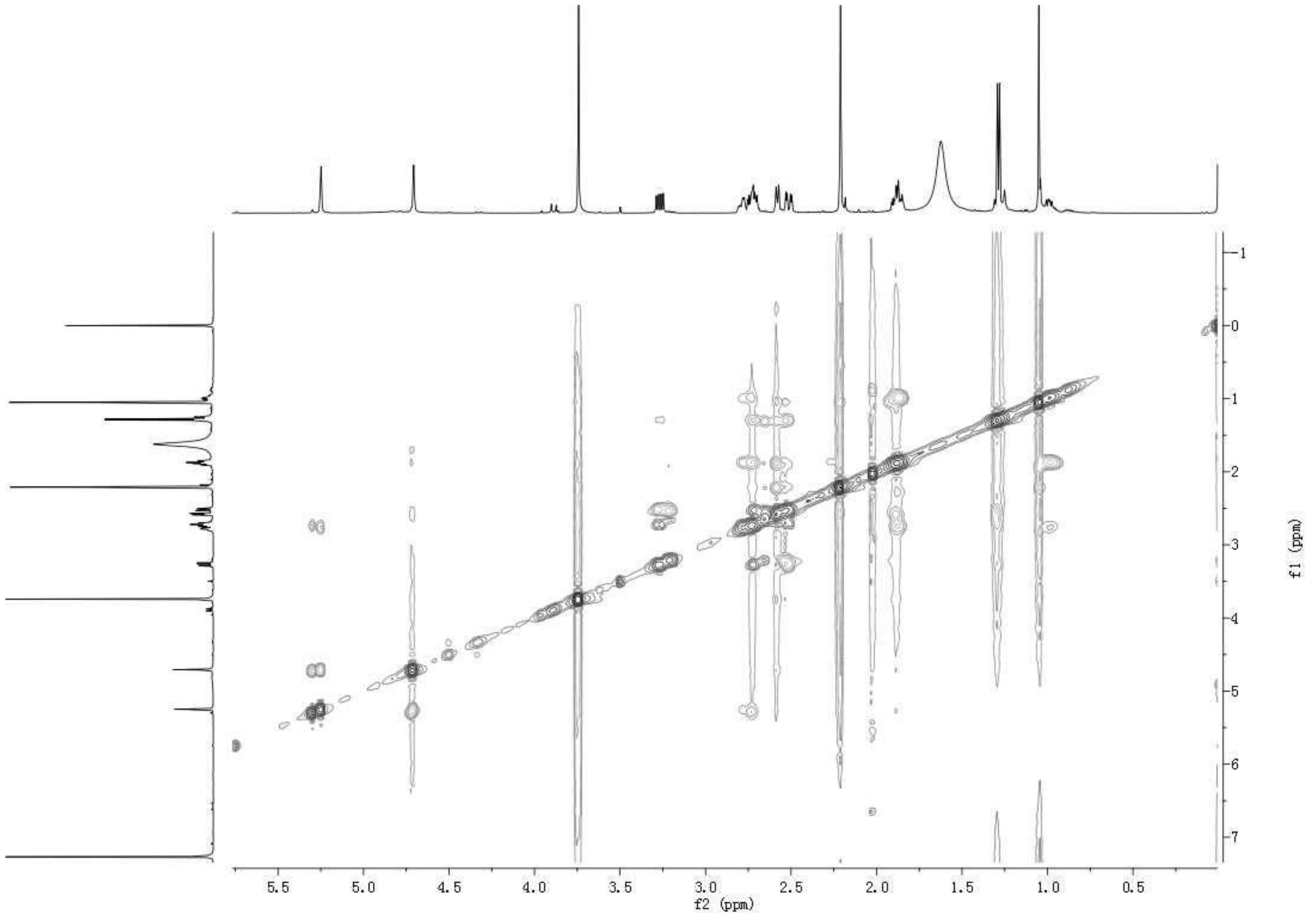

Fig. 12S ROESY spectrum of compound **2**.

Compound **3**

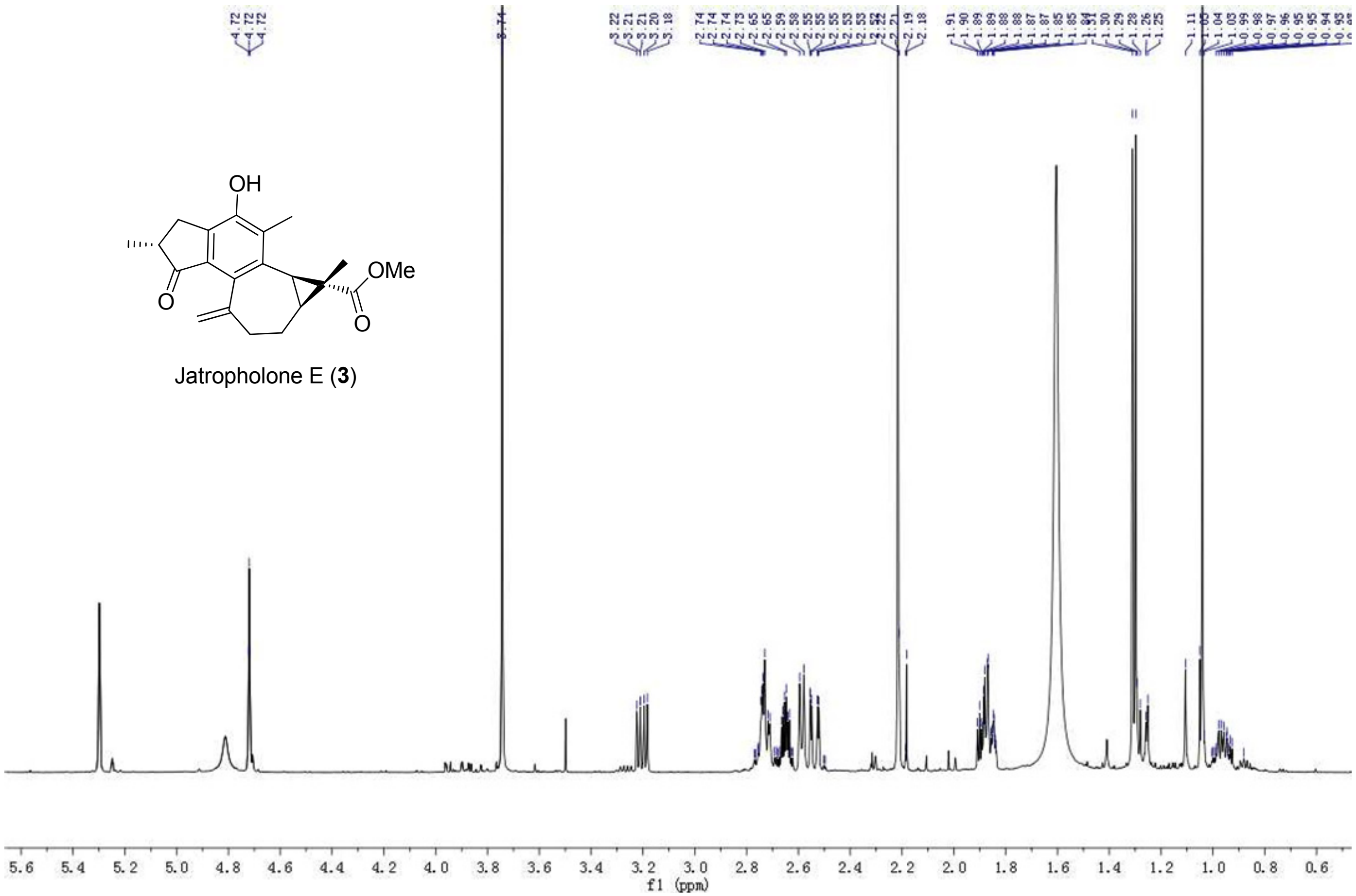

Fig. 13S ^{1}H NMR spectrum of compound **3** ($CDCl_3$, 600 MHz).

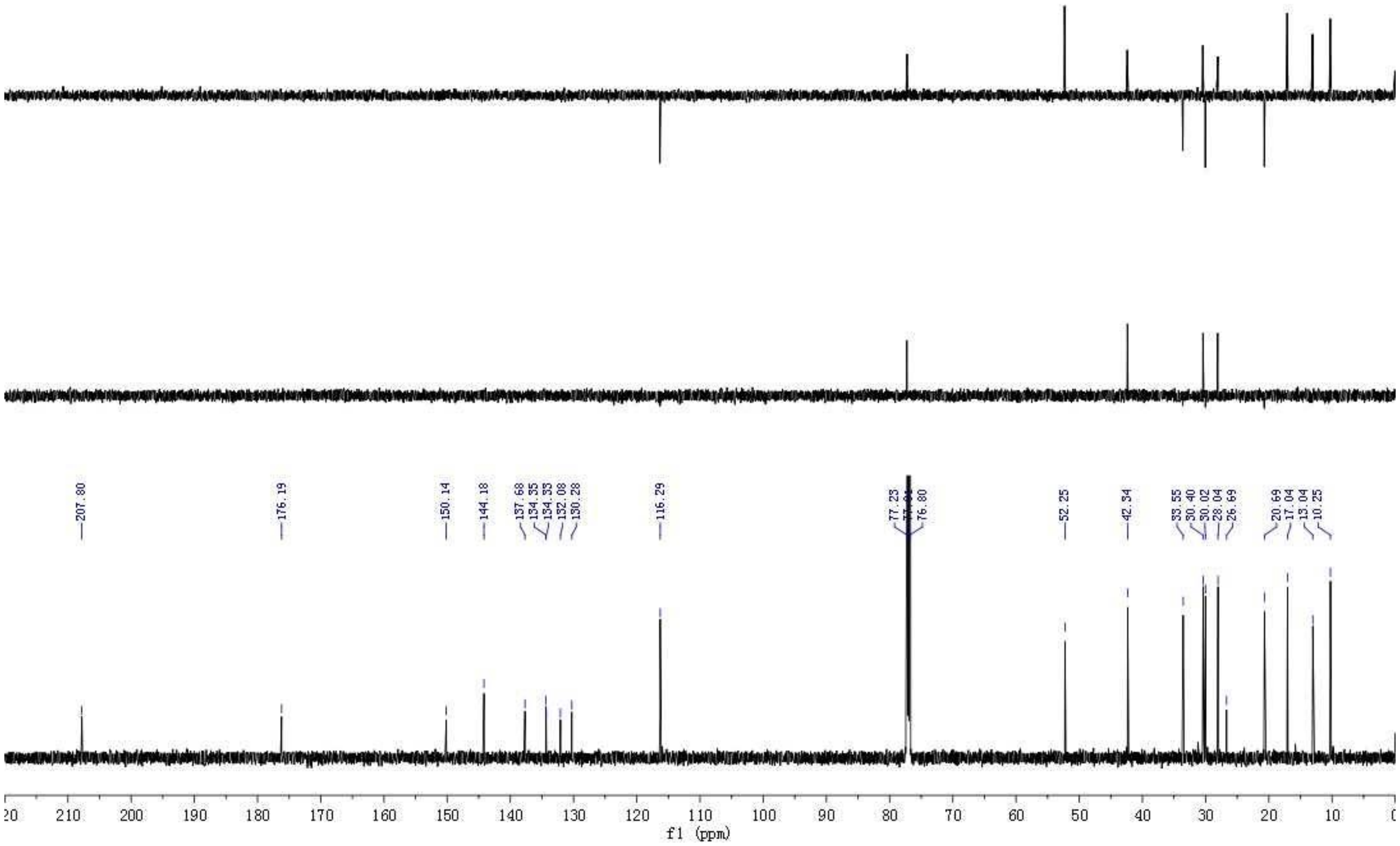

Fig. 14S ^{13}C NMR spectrum of compound **3** ($CDCl_3$, 150 MHz).

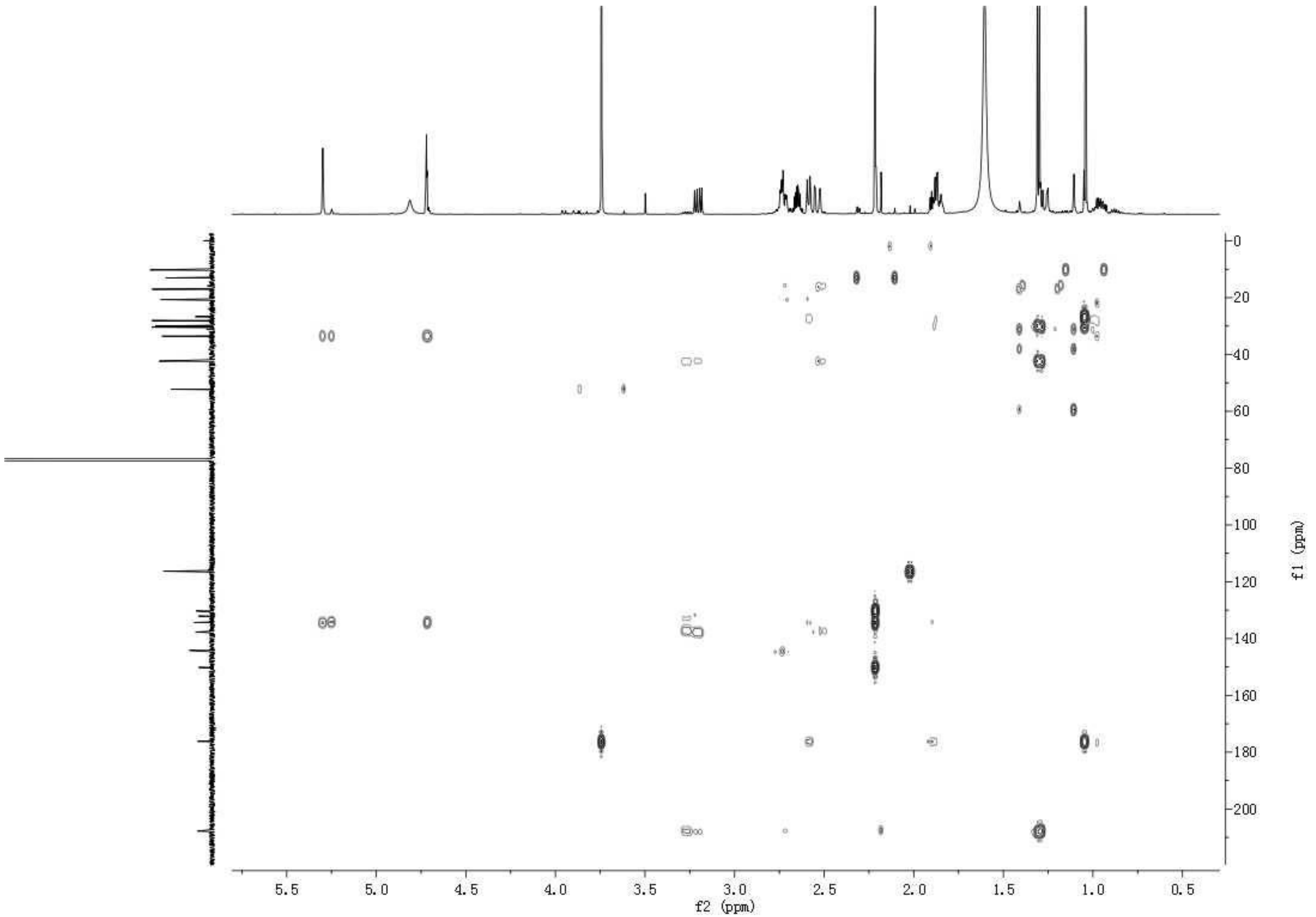

Fig. 15S HMBC spectrum of compound **3**.

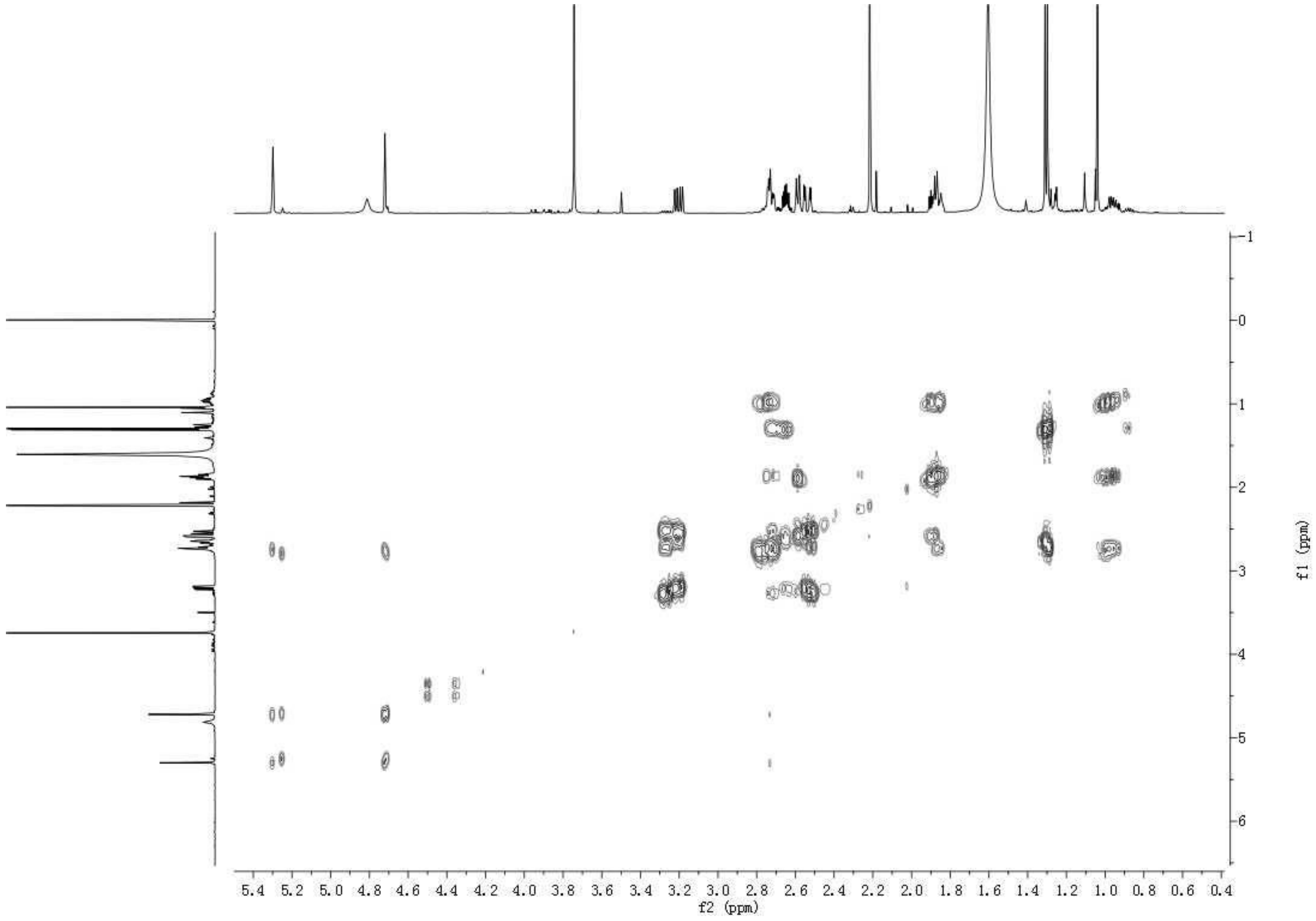

Fig. 16S ^{1}H-^{1}H COSY spectrum of compound **3**.

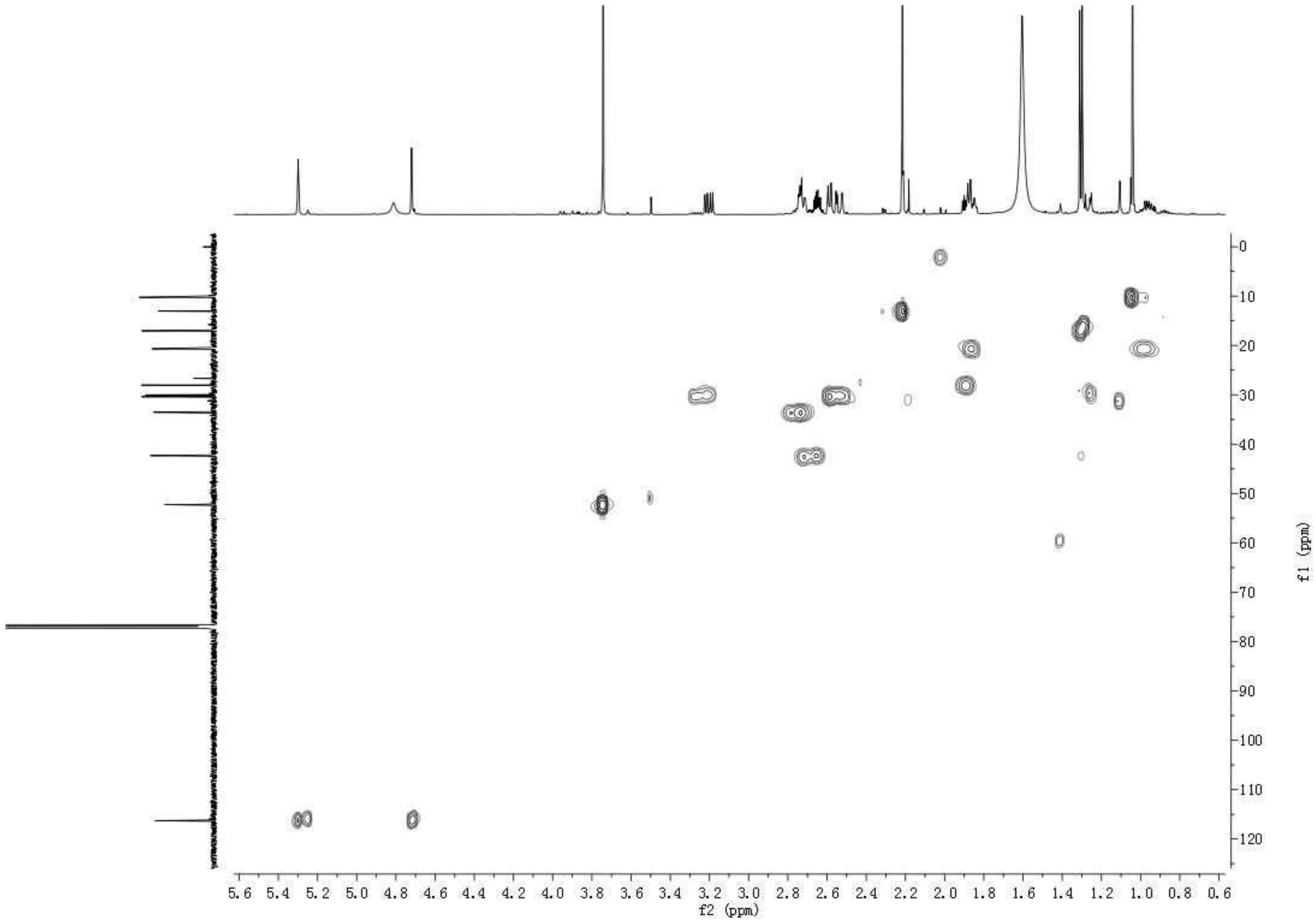

Fig. 17S HSQC spectrum of compound **3**.

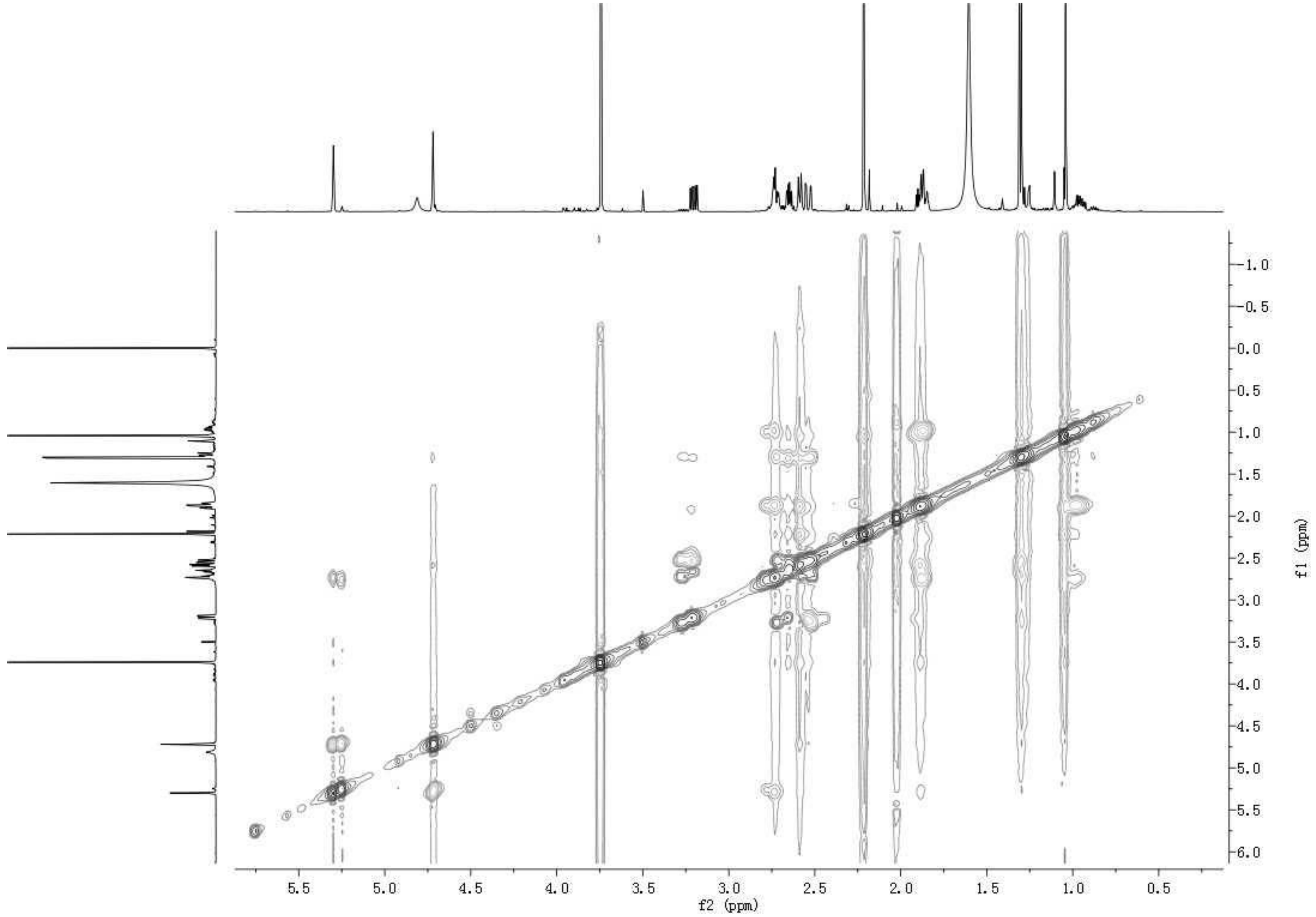

Fig. 18S ROESY spectrum of compound **3**.
